# Comparison between a clinical diagnosis method and the surveillance technique of the Center for Disease Control and Prevention for identification of mechanical ventilator-associated pneumonia

**DOI:** 10.5935/0103-507X.20150047

**Published:** 2015

**Authors:** Renata Waltrick, Dimitri Sauter Possamai, Fernanda Perito de Aguiar, Micheli Dadam, Valmir João de Souza, Lucas Rocker Ramos, Renata da Silva Laurett, Kênia Fujiwara, Milton Caldeira, Álvaro Koenig, Glauco Adrieno Westphal

**Affiliations:** 1 Intensive Care Residency Program, Hospital Municipal São José - Joinville (SC), Brazil.; 2 Physiotherapy Service, Hospital Municipal São José - Joinville (SC), Brazil.; 3 Physiotherapy Service, Centro Hospitalar Unimed - Joinville (SC), Brazil.; 4 Faculdade de Medicina, Universidade da Região de Joinville - Joinville (SC), Brazil.; 5 Hospital Infection Control Committee, Hospital Municipal São José - Joinville (SC), Brazil.; 6 Hospital Infection Control Committee, Centro Hospitalar Unimed - Joinville (SC), Brazil.; 7 Intensive Care Unit, Centro Hospitalar Unimed - Joinville (SC), Brazil.

**Keywords:** Infection, Pneumonia/diagnosis, Respiration, artificial/adverse effects, Sepsis, Intensive care units

## Abstract

**Objective:**

>To evaluate the agreement between a new epidemiological surveillance method of the Center for Disease Control and Prevention and the clinical pulmonary infection score for mechanical ventilator-associated pneumonia detection.

**Methods:**

This was a prospective cohort study that evaluated patients in the intensive care units of two hospitals who were intubated for more than 48 hours between August 2013 and June 2014. Patients were evaluated daily by physical therapist using the clinical pulmonary infection score. A nurse independently applied the new surveillance method proposed by the Center for Disease Control and Prevention. The diagnostic agreement between the methods was evaluated. A clinical pulmonary infection score of ≥ 7 indicated a clinical diagnosis of mechanical ventilator-associated pneumonia, and the association of a clinical pulmonary infection score ≥ 7 with an isolated semiquantitative culture consisting of ≥ 10^4^ colony-forming units indicated a definitive diagnosis.

**Results:**

Of the 801 patients admitted to the intensive care units, 198 required mechanical ventilation. Of these, 168 were intubated for more than 48 hours. A total of 18 (10.7%) cases of mechanical ventilation-associated infectious conditions were identified, 14 (8.3%) of which exhibited possible or probable mechanical ventilatorassociated pneumonia, which represented 35% (14/38) of mechanical ventilator-associated pneumonia cases. The Center for Disease Control and Prevention method identified cases of mechanical ventilator-associated pneumonia with a sensitivity of 0.37, specificity of 1.0, positive predictive value of 1.0, and negative predictive value of 0.84. The differences resulted in discrepancies in the mechanical ventilator-associated pneumonia incidence density (CDC, 5.2/1000 days of mechanical ventilation; clinical pulmonary infection score ≥ 7, 13.1/1000 days of mechanical ventilation).

**Conclusion:**

The Center for Disease Control and Prevention method failed to detect mechanical ventilatorassociated pneumonia cases and may not be satisfactory as a surveillance method.

## Abstract (pt)

**Objetivo:**

Avaliar a concordância entre um novo método de vigilância epidemiológica do *Center for Disease Control and Prevention* e o *Clinical Pulmonary Infection Score* para detecção de pneumonia associada à ventilação mecânica.

**Métodos:**

Coorte prospectiva que avaliou pacientes internados nas unidades de terapia intensiva de dois hospitais que permaneceram intubados por mais de 48 horas no período de agosto de 2013 a junho de 2014. Os pacientes foram avaliados diariamente pelos fisioterapeutas com o *Clinical Pulmonary Infection Score*. De forma independente, um enfermeiro aplicou o novo método de vigilância proposto pelo *Center for Disease Control and Prevention*. Avaliou-se a concordância diagnóstica entre os métodos. *Clinical Pulmonary Infection Score* ≥ 7 foi considerado diagnóstico clínico de pneumonia associada à ventilação mecânica, considerando-se diagnóstico definitivo a associação de *Clinical Pulmonary Infection Score* ≥ 7 com germe isolado em cultura semiquantitativa ≥ 104 unidades formadoras de colônias.

**Resultados:**

De 801 pacientes admitidos nas unidades de terapia intensiva, 198 estiveram sob ventilação mecânica. Destes, 168 permaneceram intubados por mais de 48 horas. Identificaram-se 18 (10,7%) condições infecciosas associadas à ventilação mecânica e 14 (8,3%) pneumonias associadas à ventilação mecânica possíveis ou prováveis, representando 35% (14/38) diagnósticos clínicos de pneumonia associada à ventilação mecânica. O método do *Center for Disease Control and Prevention* identificou casos de pneumonia associada à ventilação mecânica com sensibilidade de 0,37 e especificidade de 1,0, com valor preditivo positivo de 1,0 e negativo de 0,84. As diferenças implicaram em discrepâncias na densidade de incidência de pneumonia associada à ventilação mecânica (CDC: 5,2/1000 dias de ventilação mecânica; *Clinical Pulmonary Infection Score* ≥ 7: 13,1/1000 dias de ventilação mecânica).

**Conclusão:**

O método do *Center for Disease Control and Prevention* falhou na detecção de casos de pneumonia associada à ventilação mecânica e pode não ser satisfatório como método de vigilância.

## INTRODUCTION

Mechanical ventilator-associated pneumonia (VAP) is highly prevalent and is associated with high mortality rates.^(1)^ It is estimated that the incidence of VAP increases according to the number of days on mechanical ventilation (MV), representing one of the main infectious complications of the critically ill.^(2)^ Although controversial, the incidence of VAP has been used as an indicator of the quality of care in intensive care units (ICU) because it is a potentially preventable condition.^(3)^ The VAP rates reported by many hospitals have declined, and many institutions have been reporting no VAP cases in recent years.^(4)^ However, Dalmora et al. showed that this decrease has not been followed by a corresponding reduction in antibiotic use, leading them to question the reliability and accuracy of the diagnostic criteria used.^(5)^ Ego et al. demonstrated that the concomitant application of different diagnostic criteria in the same population of critically ill patients resulted in a large discrepancy in VAP incidence rates, ranging from 4 - 42%, depending on the criteria used. At the same time, the use of more stringent criteria delayed the diagnosis by four days, which was reflected by an increase in mortality from 50% to 80%.^(3)^ This variability in the recognition and incidence of VAP may be explained by the subjectivity and lack of uniformity in the application of certain diagnostic criteria, such as chest X-ray changes and characterization of airway secretions.

In 2013, the National Healthcare Safety Network/Center for Disease Control and Prevention (NHSN/CDC) published a new surveillance protocol to standardize VAP confirmation criteria and thereby increase the reliability of indicators in different institutions.^(6)^ The NHSN/CDC 2013 method advocates the evaluation of the partial pressure of oxygen/fraction of inspired oxygen ratio (PaO_2_/FIO_2_) over time and excludes chest X-ray interpretation and characterization of tracheal aspirates. This method leads to a reduction in the potential for human participation bias in diagnosis and yields greater inter-observer and inter-institutional agreement.^(6,7)^ Despite these efforts, recent studies have shown low agreement between clinical and surveillance methods,^(8,9)^ and the NHSN/CDC 2013 method exhibits low sensitivity in identifying VAP cases that have been detected clinically.^(8)^ The practical implications of a surveillance method that underestimates the clinical diagnosis are that it can produce a false impression of lower VAP rates, and hence, VAP prevention improvement measures may not be implemented because it appears that the rates are being controlled.

In this context, the objective of the present study was to evaluate the agreement between bedside clinical evaluation and the NHSN/CDC 2013 surveillance method in the identification of cases of VAP.

## METHODS

This prospective observational study was performed in two general ICU with a total of 18 clinical and surgical beds. All patients older than 18 years of age who remained on MV for more than 48 consecutive hours from August 1, 2013 to June 30, 2014 were included. Patients who did not complete 48 hours of MV or who were younger than 18 years of age were excluded. The study was approved by the Ethics Committee of the participating institutions (*Hospital Municipal São José* and *Centro Hospitalar Unimed*) under protocol number CAAE 20559613.1.0000.5362. Given the observational nature of the study, informed consent of the patients was not required.

Institutional VAP prevention protocols (established since 2010) were applied daily to all patients and included the following procedures: head elevated to 30º, gastric ulcer prophylaxis, deep vein thrombosis prophylaxis, daily sedation interruption, and oral hygiene with chlorhexidine.^(10,11)^

Daily samples were taken of all variables that allowed VAP identification, both for the clinical method and the surveillance protocol (NHSN/CDC 2013). The data required for both two methods were collected concomitantly by different professionals who did not exchange information regarding their findings. The NHSN/CDC 2013 protocol variables (Table 1) were recorded prospectively by Hospital Infection Control Commission nurses. At the same time, physical therapists recorded the variables used to perform the clinical VAP diagnosis. To decrease variability in interpreting clinical variables, we used the clinical pulmonary infection score (CPIS), and a CPIS ≥ 7 was considered to be a clinical diagnosis of VAP (Table 2).^(12-14)^ The CPIS obtained from the evaluation by the physical therapist was always validated by an intensive care physician. All professionals involved in data collection received specific training.

**Table 1:** National Healthcare Safety Network/Center for Disease Control and Prevention definitions

**Concept**	**Definition**
Period of stability	2 or more consecutive days of maintenance or decrease in the FIO_2_ or PEEP
VAC	After a stabile period, the need to increase the PEEP ≥ 3cmH_2_O or the FIO_2_ ≥ 20% for 2 consecutive days.
iVAC	VAC + temperature > 38ºC or < 36ºC OR Leucocytes > 12000/mm^3^ or < 4000/mm^3^ AND administration of a new antibiotic for 4 or more consecutive days
Possible VAP	iVAC + purulent respiratory secretions (≥ 25 neutrophils/field and ≤ 10 squamous cells/field) OR positive mini-BAL culture
Probable VAP	iVAC + purulent respiratory secretions (≥ 25 neutrophils/field and ≤ 10 squamous cells/field) AND positive mini-BAL culture

FIO_2_ - fraction of inspired oxygen; PEEP - positive end expiratory pressure; VAC - ventilatorassociated complication; iVAC - infection-related ventilator-associated complication; mini-BAL - mini-bronchoalveolar lavage; VAP - mechanical ventilator-associated pneumonia.

**Table 2:** Clinical pulmonary infection score definitions

**Variable**	**Score**
Temperature (ºC)	
36.5 - 38.4	0
38.5 - 39	1
< 36 or > 39	2
Leucocytes x 1000 (cells/mm^3^)	
4 - 11	0
11 - 17	1
> 17	2
Tracheal secretion (amount)	
Low	0
Moderate	1
Abundant	2
Purulent	+ 1
PaO_2_/FIO_2_	
> 250	0
< 250	2
Chest X-ray (infiltrate aspect)	
Clean	0
Diffuse	1
Localized	2

PaO_2_/FIO_2_ - partial pressure of oxygen/fraction of inspired oxygen.

For microbiological confirmation of VAP, airway secretion samples were collected using the blind mini-bronchoalveolar lavage (mini-BAL) technique. This technique involves aseptically inserting a suction catheter through the tracheal tube, introducing 20mL of saline, and applying immediate suction without pulling the catheter out. The samples were transferred to sterile vials, labeled, kept at room temperature, and immediately sent to the laboratory. Samples with bacterial growth ≥ 10^4^ colony-forming units per milliliter (CFU/mL) were considered positive. All samples were processed in a standardized manner and subjected to cytological and quantitative microbiological analysis.^(15)^

The following variables were analyzed: age, gender, the Acute Physiology and Chronic Health Evaluation II (APACHE II) score, length of ICU stay, MV time, and mini-BAL isolated bacteria. Variables with a scatter histogram with a normal curve overlay, similarity between the mean and median, and kurtosis between -1 and 1 were evaluated. Variables with a normal distribution are presented as the means and standard deviations, and non-symmetrical variables are presented as the medians and interquartile ranges. The chi-square test was used to compare categorical variables expressed in absolute and relative values. Because lung tissue samples for microbiological analysis could not be obtained, we considered the occurrence of a CPIS ≥ 7 associated with a positive mini-BAL culture the gold standard in defining sensitivity and specificity.^(16)^ The agreement between the methods was evaluated using Cohen’s kappa test. P values < 0.05 were considered statistically significant. Data were analyzed using IBM Statistical Package for Social Sciences (SPSS) version 19 and Stata 11.0.

## RESULTS

Over the course of one year, 801 patients were admitted to the study ICUs, and 198 patients required MV. Of these, 168 remained intubated for more than 48 hours and were included in the analysis. The demographic characteristics of patients with VAP are shown in table 3.

**Table 3:** Characteristics of patients with mechanical ventilator-associated pneumonia

	**Clinical diagnosis (CPIS) (N = 38)**	**NHSN/CDC (2013) (N = 14)**
Age (years)	54.2 ± 19.1	56.7 ± 20.1
Gender		
Male	24 (63.2)	7 (50)
Female	14 (36.8)	7 (50)
APACHE II score	21.4 ± 8.0	20.6 ± 8.9
ICU time (days)	26.9 ± 16.6	30.3 ± 21.5
MV time (days)	21.5 ± 15.2	21.8 ± 15.9

CPIS - clinical pulmonary infection score; NHSN/CDC - National Healthcare Safety Network/Center for Disease Control and Prevention; SD - standard deviation; APACHE II - Acute Physiology and Chronic Health Evaluation II; ICU - intensive care unit; MV - mechanical ventilation. The results are expressed as number (%) or as the mean ± standard deviation.

**Figure 1:**
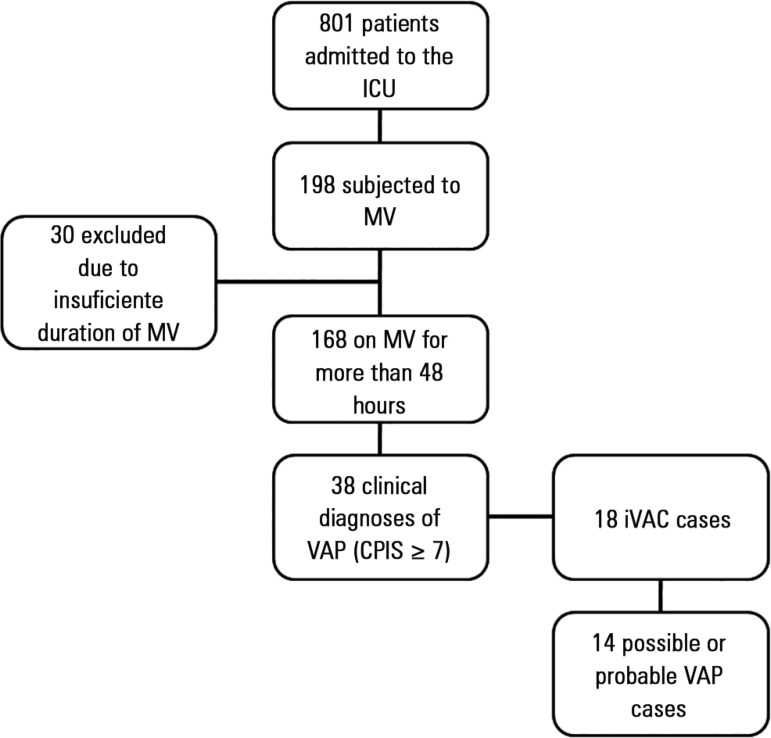
Flowchart of patient inclusion over the course of the study. ICU - intensive care unit; MV - mechanical ventilation. CPIS - clinical pulmonary infection score; VAP - mechanical ventilator-associated pneumonia; iVAC - infection-related ventilator-associated complications.

A total of 38 clinical diagnoses of VAP were obtained using the CPIS, resulting in a VAP incidence density of 13.1/1000 MV days. The NHSN/CDC 2013 surveillance method identified 14 VAP cases, corresponding to a VAP incidence density of 5.2/1000 MV days. All cases of VAP identified by the NHSN/CDC 2013 method had a CPIS of ≥ 7. The patient inclusion flowchart is shown in figure 1.

A total of 18 (10.7%) cases of infection-related ventilator-associated complications (iVAC) were identified, of which 14 (8.3%) were possible or probable VAP, representing 35% (14/38) of the clinical diagnoses of VAP as determined by the CPIS. Moderate agreement was observed between the clinical diagnosis and surveillance (NHSN/CDC 2013) methods in identifying VAP cases (kappa = 0.47; 95% confidence interval [95% CI]: 0.44 - 0.91; p < 0.001). The NHSN/CDC 2013 method had a sensitivity of 0.37 and a specificity of 1.0 for identifying cases of VAP, compared to the clinical diagnosis using a CPIS ≥ 7.

When positive mini-BAL culture results along with a CPIS ≥ 7 were used to define the VAP diagnosis, the kappa agreement coefficient was 0.63. Under this condition, the NHSN/CDC 2013 method had a sensitivity of 0.58 and a specificity of 0.84.

A predominance of Gram-negative bacilli was observed among the mini-BAL isolated pathogens (Table 4).

**Table 4:** Pathogens isolated in the mini-bronchoalveolar lavage culture

**Pathogen N (%)**	**Clinical diagnosis (CPIS) (N = 38)**	**NHSN/CDC 2013 (N = 14)**
*Staphylococcus aureus*	5 (13.1)	3 (21.4)
*Enterococcus faecalis*	1 (2.6)	1 (7.1)
*Klebsiella spp*	6 (15.7)	5 (33.3)
*Haemophilus spp*	3 (7.8)	2 (14.3)
*Escherichia coli*	3 (7.8)	2 (14.3)
*Pseudomonas spp*	4 (10.5)	1 (7.1)
*Acinetobacter spp*	2 (5.2)	-
*Proteus mirabilis*	1 (2.6)	1 (7.1)
Candida spp*	2 (5.2)	2 (14.3)
Negative cultures	16 (42.1)	3 (21.4)

CPIS - clinical pulmonary infection score; NHSN/CDC - National Healthcare Safety Network/Center for Disease Control and Prevention.

* In all cases that *Candida spp* was detected, an isolated bacterium with more than 10^4^ CFU/mL was present.

## DISCUSSION

Our results showed a decreased ability of the NHSN/CDC 2013 surveillance method to identify patients with a clinical diagnosis of VAP, suggesting a large discrepancy in determining the VAP incidence density. Similarly, previous studies have shown poor agreement between the clinical diagnosis of VAP and surveillance methods proposed by the CDC.^(7-9,16)^

When comparing a previously used NHSN/CDC surveillance method (prior to 2013) with the clinical diagnosis method, Skrupky et al.^(16)^ showed that only 14.5% of clinically diagnosed cases were identified using the surveillance method. The incidence density decreased from 8.5 to 1.2 cases per 1,000 MV days, and the kappa agreement coefficient was 0.26. Despite using the current version of the NHSN/CDC method (2013), our findings are very similar to those presented by Skrupky et al. We observed that only 35% of clinical diagnoses were confirmed by the NHSN/CDC 2013 method; this result was reflected by very different VAP incidence densities (13.1 cases/1,000 MV days versus. 5.2/1000 MV days). In 2006, a study conducted in a trauma ICU revealed that diagnosis of VAP according to the surveillance method used at that time resulted in the non-treatment of VAP in 16% of patients.^(8)^

However, when those authors added the respiratory secretion culture result to the clinical diagnosis of VAP, they found an increase in agreement with the surveillance methods used at that time.^(8,14)^ Skrupky et al.^(16)^ obtained microbiological confirmation of VAP in 88% of clinical diagnoses and in 92% of cases using the former surveillance method, while Miller et al.^(8)^ observed similar VAP incidence density rates (clinical, 34/1000 MV days versus surveillance, 36/1000 MV days). Similarly, when comparing VAP clinical diagnoses associated with microbiological results to the NHSN/CDC 2013 method, we also observed an increase in agreement between the methods for VAP identification. These findings suggest that surveillance methods may underestimate bedside clinical diagnoses and that the combined use of stricter methods, such as microbiological cultures, can improve the clinical accuracy of the method.

The retrospective diagnosis of VAP follows a mandatory sequential flow of pathophysiological changes that lead to a diagnosis. Thus, the low sensitivity of the older surveillance methods observed by Skrupky et al.^(16)^ could be attributed to the retrospective nature of the VAP diagnosis, in contrast to the clinical bedside diagnosis, which is performed in real time.

In an attempt to improve the performance of the VAP surveillance method, the NHSN/CDC 2013 method requires the prospective collection of clinical variables that define the VAP diagnosis. Despite daily and prospectively constructing the surveillance spreadsheet suggested by the NHSN/CDC 2013 method and despite collection being concurrent to the daily clinical evaluation using the CPIS, we found no performance improvement in the surveillance method compared to previous studies. A clear moderate agreement (kappa = 0.47) resulted from the low sensitivity in relation to the CPIS (sensitivity = 0.37 and specificity = 1.0). These results replicate the findings of a recent study by Lilly et al.^(9)^ involving 8,408 patients on MV who were characterized by the prospective and electronic collection of clinical variables stipulated by the NHSN/CDC 2013 method. The sensitivity of this method compared to the clinical diagnosis was 0.32, and the specificity was 0.96.

The low sensitivity of the NHSN/CDC 2013 method for detecting VAP may be related to the different assumptions of the method, such as the very high positive end-expiratory pressure (PEEP) and FIO_2_ cut-off points when considering ventilation deterioration and the mandatory period of 48 hours of MV stability, which cannot be observed in all patients with VAP.

Originally, changes in chest X-rays and in tracheal secretions were a fundamental and early part of VAP diagnosis. However, the NHSN/CDC 2013 method excluded these subjective variables. The exclusion of high sensitivity variables could affect the accuracy of the method and explain the low sensitivity found in the present study and corroborated by Lilly et al.^(9)^ Because surveillance methods are not intended for bedside diagnosis, nonspecific variables usually considered in the diagnosis of infection, such as glycemic change and dysfunction of systems other than the respiratory system, are not included. The omission of these variables may contribute to the difference between the methods presently studied.

This study has some limitations that should be taken into consideration. Although the results corroborate other publications on the subject, the sample size and the small number of participating centers limited the study power. The study was originally designed to include four ICU, but operational problems resulted in limiting the centers to two units. The use of a gold standard to evaluate VAP diagnosis presents a great practical difficulty because it requires pulmonary tissue cultures to be performed.^(14)^ For this reason, and as in other studies,^(8,9,14,16)^ we compared the surveillance method to a clinical laboratory method (CPIS ≥ 7 plus mini-BAL culture > 10^4^ CFU/mL), using the latter as our reference standard. Thus, because we did not use a gold standard, the process by which we defined the sensitivity and specificity of the method is open to criticism. We must also consider the use of the CPIS to standardize clinical diagnoses as a limitation because, in spite of it being one of the best VAP clinical diagnosis methods available, it has low diagnostic accuracy.^(12-14)^

## CONCLUSION

The greatest implication of our findings was the identification and confirmation of the weakness of the *National Healthcare Safety Network/Center for Disease Control and Prevention* 2013 method as an inter-institutional comparison method for the incidence of mechanical ventilator-associated pneumonia because the method did not lead to the detection of clinically diagnosed ventilator-associated pneumonia cases and therefore may not be suitable as a surveillance method.
